# Baixiangdan capsule and Shuyu capsule regulate anger-out and anger-in, respectively: GB1–mediated GABA can regulate 5-HT levels in multiple brain regions

**DOI:** 10.18632/aging.204589

**Published:** 2023-03-21

**Authors:** Xiaoju Liu, Haijuan Wang, Xiaoyu Wang, Yinxia Ning, Wei Liu, Jie Gao

**Affiliations:** 1 Shandong University of Traditional Chinese Medicine, First Clinical Medical College, Jinan, China; 2 Shandong Cancer Hospital and Institute, Shandong First Medical University and Shandong Academy of Medical Sciences, Jinan, China; 3 Shandong University of Traditional Chinese Medicine, College of Traditional Chinese Medicine, Jinan, China; 4 Department of Encephalopathy, Shandong University of Traditional Chinese Medicine Second Affiliated Hospital, Jinan, China; 5 Shandong University of Traditional Chinese Medicine, Office of Academic Research, Jinan, China

**Keywords:** anger-out, anger-in, baixiangdan capsule, shuyu capsule, serotonin, GABABR1

## Abstract

The identity of the mechanism by which the Baixiangdan capsule (BXD) and the Shuyu capsule (SY) control anger-out (AO) and anger-in (AI) in rodents is unclear. The current study clarified the intervention role of BXD and SY on AO and AI male rats. We further explored the differences between BXD and SY in the treatment of AO and AI rats. Social isolation combined with the resident-intruder paradigm was used to establish the anger-out and AI rats models. On this basis, GABA content in the dorsal raphe nucleus (DRN) and serotonin (5-HT) contents in these brain regions were detected using ELISA after various time courses (0, 1, 3, 5, and 7 days) treated with BXD and SY. Co-expression of 5-HT and GB1 in the DRN was detected. GB1-specific agonist baclofen and GB1-specific inhibitor CGP35348 were injected into the DRN. Changes in 5-HT levels in these brain regions were then detected. After treatment, rats in the BXD group exhibited lower aggressive behavior scores, longer latencies of aggression, lower total distances in the open field test, and a higher sucrose preference coefficient. Meanwhile, rats in the SY group exhibited higher aggressive behavior scores, shorter latencies of aggression, higher total distances in the open field test, and higher sucrose preference coefficients. With increasing medication duration, 5-HT levels in these brain regions were increased gradually, whereas GABA levels in the DRN were decreased gradually, and all recovered to normal levels by the 7th day. A large number of 5-HT-positive cells could be found in the immunofluorescence section in the DRN containing GABABR1 (GB1)-positive cells, indicating that 5-HT neurons in the DRN co-expressed with GB1. Furthermore, after the drug intervention, the 5-HT level in the DRN was elevated to a normal level, and the GB1 level in the DRN was decreased to a normal level. After the microinjection of baclofen into the DRN, the 5-HT contents in these brain regions were decreased. By contrast, the 5-HT contents were increased after injection with CGP35348. BXD and SY could effectively improve the abnormal behavior changes of AO and AI rats, and the optimal duration of action was 7 days. The improvement way is as follows: Decreased abnormal increase of GABA and GB1 in the DRN further mediated synaptic inhibition and increased 5-HT level in the DRN, leading to increased 5-HT levels in the PFC, hypothalamus, and hippocampus. Therefore, GB1-mediated GABA in the DRN could regulate 5-HT levels in these brain regions, which may be one of the ways by which BXD and SY treat AO and AI, respectively.

## INTRODUCTION

Anger is a normal human emotional response when encountering various unacceptable situations [1]. Ultimately, anger is a subjectively intolerable emotion [2]. In extreme or long-time suffering, anger may lead to aggressive behavior, hostility, violence, anxiety, and adverse health consequences [3]. Anger is probably one of the most controversial basic emotions because of its difficulty to spot during human development [4]. Anger is defined as a negative emotional reaction to others’ obstruction and unfair behaviors [5]. Based on the differences in personal characteristics and enduring propensity [6], anger typically expresses itself in two opposing ways: anger-in (AI) and anger-out (AO) [7]. AO is defined as a subjective feeling, classified as “anger directed outward away from the self”, whereas AI is defined as a subjective feeling, classified as “anger directed toward the self” [8]. According to traditional Chinese medicine, anger is associated with the liver, which is responsible for calming emotions. The liver loses catharsis, too much and too little. Too much liver catharsis can lead to the inversion of liver qi, which is manifested as irritability, excitement, anger, and venting; this is the characteristic of AO. Insufficient liver catharsis can lead to stagnation of liver qi, which is manifested as depression and anger but unable to vent; this is the characteristic of AI. AO means to vent your anger toward another person or something else, and AI means to vent your anger without unleashing it, to keep it in your mind, to direct it toward yourself. When treating anger, traditional Chinese medicine pays attention to syndrome differentiation and treatment.

The Baixiangdan capsule (BXD) and the Shuyu capsule (SY) are the new capsule formulation that combines several plant extracts. BXD is the refined prescription of Jingqianping granules (derived from Chaihu Shugan Powder), and SY is the refined prescription of Jingqianshu granules (derived from Xiaoyao Powder). Jingqianping and Jingqianshu granules can be used to treat two different syndrome types of premenstrual syndrome–liver-qi invasion and liver-qi depression, respectively. These granules can alleviate their typical symptoms: irritability and anger (Jingqianping granule), and depression (Jingqianshu granule); their clinical efficacy is significant [9–12]. BXD–the main active ingredients are paeoniflorin, paeonol, and alpha-cyperone [13–15] is composed of Baishao (*Radix Paeoniae Alba.*), Xiangfu (*Cyperi Rhizoma*), and Danpi (*Moutan Cortex*), which may have antipyretic, anti-inflammatory, analgesic, and neuroprotective functions [16, 17]. BXD has the effect of soothing the liver, regulating qi, removing distension, and relieving pain. It is a drug for liver qi inversion and AO treatment. SY–the main effective components are bupleurum, paeoniflorin, and volatile oil of *Rhizoma cyperus*–is composed of Chaihu (*Bupleuri Radix*), Xiangfu (*Cyperi Rhizoma*), Baishao (*Radix Paeoniae Alba.*), and Gancao (*Glycyrrhizae Radix et Rhizoma*), which can alleviate depressive symptoms, and the mechanism mainly focuses on specific brain regions [18, 19]. SY has the effect of soothing the liver, relieving depression, nourishing blood, and softening the liver. It is a drug for liver qi stagnation and AI. Paeoniflorin has many biological effects: enhancement of cognitive ability, improvements in learning disabilities, and nerve protection [20]. BXD can reduce the aggressive behavior score and the total open field score of AO rats and can improve the sugar water preference coefficient and body mass, to reduce the aggressiveness of AO rats, calm their irritability and tension symptoms, and improve the expression of AO emotions [21]. Furthermore, BXD has entered the third phase of clinical research, and the clinical report has significant advantages in relieving liver-qi inversion, which can effectively relieve PMS syndrome of liver-qi inversion [22, 23]. SY is an effective antidepressant treatment. Clinical studies have reported that SY is as effective as fluoxetine in anti-depression [24–26]. In conclusion, BXD and SY can effectively relieve anger and depression, but the neural effects underlying these actions are unclear.

The emotion regulation circuit includes several brains regions, including the prefrontal cortex (PFC), hypothalamus, hippocampus, amygdala, anterior cingulate cortex, ventral striatum, insular cortex, and other interconnected structures [27]. Studies have demonstrated that defensive rage or aggression can typically be elicited by electrical stimulation of hypothalamic sites in cats and rats [28]. The degree of anger expression in pictures or sounds was positively correlated with the signal intensity of bloodstreams and glucose metabolism in the prefrontal and hippocampal [29]. The activity in the ventromedial prefrontal cortex preferentially responded to anger expressions oriented to self [30]. Thus, the PFC, hippocampus, and hypothalamus play a crucial role in anger regulation [31–33].

Numerous studies have demonstrated that various neurotransmitters and hormones, including serotonin (5-HT), norepinephrine, dopamine, androgens, and estrogens play a role in anger regulation, with 5-HT being the most important [34, 35]. Major serotonergic populations contained in the midbrain dorsal raphe and median raphe nuclei, then projecting to various regions of the brain such as the PFC, hippocampus, and hypothalamus [36, 37], participate in regulating cognition, intuition, and emotion [38]. The study demonstrated that depressed patients who experience anger episodes may have relatively greater serotonin dysregulation than depressed patients without these episodes [27]. However, little is known about how the 5-HT system was involved in the expression of various forms of anger. GABA is an important inhibitory neurotransmitter in the central nervous system [39]. Approximately 50% of synapses in the central nervous system use GABA as the neurotransmitter, which severely affects the function of the PFC [40]. GABAB receptor–a metabolic G protein-coupled receptor–is composed of GABABR1 (GB1) and GABABR2 (GB2). GB1 is responsible for binding to GABA [41, 42] and plays an essential role in maintaining normal brain function, whereas GB2 is responsible for G protein coupling [43]. GABABR1a knockout mice are more likely to suffer from stress and pleasure loss and social escape behavior [44]. The up-regulation of GABABR1a expression in the dorsal raphe nucleus (DRN) of socially isolated mice is closely related to abnormal behavior caused by social stress [45]. Thus, 5-HT, GABA, and GB1 play an essential role in anger and depression, and the specific mechanism must be further investigated.

No single structure or neuron group encodes anger in the brain. Little is known about how anger circuits connect to these brain regions and how BXD or SY relieves AO or AI. Animal (typically rodent) models have been used to investigate its anxiolytic, anti-anger, and anti-depression effects. Based on the existing studies and our team’s research, we hypothesized that BXD and SY would also be effective treatments for irritability, which can trigger impulsive and/or aggression. This finding suggests that BXD or SY can effectively alleviate AO or AI, which may be through this pathway: GB1 mediated GABA in the DRN regulates the 5-HT levels in the PFC, hypothalamus, and hippocampus.

To study the intervention effects of BXD and SY on anger-out and anger-in, we adopted a variety of modern biological technologies and used experimental rat models (AO and AI rat models were prepared based on widely recognized social isolation pressure and in combination with the resident-intruder paradigm [46, 47]).

## MATERIALS AND METHODS

### Preparations of BXD and SY

BXD (201401011, Qingdao Haichuan Innovative Biological Natural Medicine Research Center, Qingdao, China. https://www.11467.com/qingdao/co/111873.htm) – the main active ingredients are paeoniflorin, paeonol, and alpha-cyperone [13–15] is composed of Baishao (*Radix Paeoniae Alba.*), Xiangfu (*Cyperi Rhizoma.*), and Danpi (*Moutan Cortex*). SY (201401020, Qingdao Haichuan Innovative Biological Natural Medicine Research Center, Qingdao, China. https://www.11467.com/qingdao/co/111873.htm) – the main effective components are bupleurum, paeoniflorin, and volatile oil of Rhizoma cyperus–is composed of Chaihu (*Bupleuri Radix*), Xiangfu (*Cyperus rotundus L.*), Baishao (*Radix Paeoniae Alba.*), and Gancao (*Glycyrrhizae Radix et Rhizoma.*). HPLC-ESI-MSn was used to identify and assess the quality of chemical compounds in SY and quality control of SY [48], whereas HPLC was used to identify chemical compounds in BXD [23].

### Animals, groups, and treatments

Sixty male rats (Wistar, 180–220 g), and twelve male rats (Sprague-Dawley, 120–150 g) were obtained from Charles River Laboratories (Beijing, China, License No.: SCXK (Beijing) 2012–0001). The two kinds of rats were housed separately under the same conditions (temperature: 22 ± 2°C, humidity:55 ± 10%, noise ≤60dB, and 12 hours of light/dark cycle (lights on at 8:00 p.m.)), with food and water ad libitum. All behavioral tests were conducted in dim red light (<2lux). All procedures in this study strictly adhere to the NIH Guidelines for the Care and Use of Laboratory Animals. Animal experimental designs have been approved by the experimental animal ethics committee of Shandong University of Traditional Chinese Medicine (DWSY20170301).

**Figure 1:**
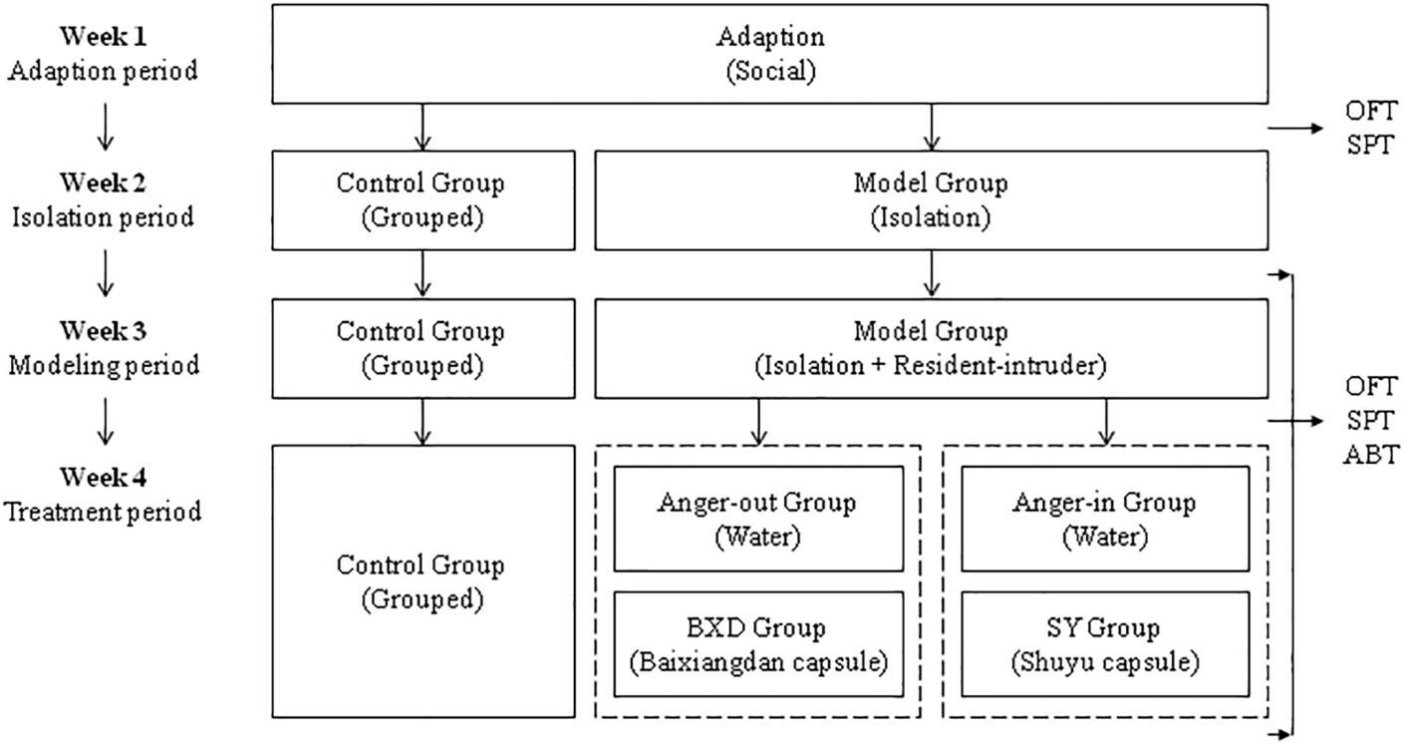
**Graphic experimental timeline.** Model Group: Different from the control group, including anger-out group and anger-in group. Abbreviations: OFT: Open field test; SPT: Sucrose preference test; ABT: Aggressive behavior test.

First, we established the rat model based on the widely recognized social isolation stress combined with the resident-intruder paradigm [47]. Wistar rats were used as residents, whereas SD rats as intruders. As shown in Figure 1, the experimental process was 4 weeks. All rats were fed adaptively (food and water ad libitum to suit the environment, 12 hours light/dark cycle for 1 week (lights on at 8:00 p.m., four rats per cage, with no manipulation except daily grasping). From the beginning of the second week, 12 Wistar rats were randomly selected as the control group with 4 per cage, and the remaining Wistar rats as the model group (AO group and AI group) with 1 per cage, and were maintained for 7 days. In the third week, rats in the model group were exposed to social isolation and resident-intruder stress every day. The SD rats were placed in the cage of the model group for 15 min. The resident was confronted with a different intruder each day based on the Latin Square design [49]. At the end of the third weekend, 48 rats in the model group were divided into the AO group (24 rats) and the AI group (24 rats) according to the comprehensive attack score of median analysis. Simultaneously, 12 rats from the AO group were randomly selected as the anger-out BXD group, and 12 rats from the AI group were randomly selected as the Anger-in SY group (12 rats in each group). In the fourth week, social isolation and resident-intruder stress still existed. Besides, rats in the BXD group were orally administered BXD suspension at a dose of 1.33 g/kg (equivalent to 8 times of clinical dose of humans) [21, 50], continuing for 7 days. Rats in the SY group were orally administered SY suspension with the same dosage and course of treatment. The control, AO, and AI groups were orally administered an equal volume of sterilized water and the same course of treatment. The sucrose preference test (SPT) and open field test (OFT) were conducted at the end of each week, and the aggressive behavior test (ABT) was conducted only at the end of the second, third, and fourth weeks.

### Behavioral evaluation

#### Aggressive behavior test (ABT)

ABT has become the most common method for studying animal depression and aggressive behavior [51]. Although aggressive behavior induced by social isolation combined with the resident-intruder paradigm is not technically defined as anger, it is most similar to anger [52]. In a sense, the generation of anger is the preparation response to an attack [53]. Aggressive behavior– the typical behavior and core symptom of anger emotion [54] is considered the standard of anger emotion induction [55].

In this test, an intruder rat was placed into the cage of a resident rat, and then observed and recorded with a camera for 15 min. After the test, the observers watched the video, and the “rodent aggression analysis method and device”, which obtained a national invention patent (Chinese patent: CN106472348A, 2017-03-08), was used to calculate the aggression behavior score. This can effectively enhance the accuracy and objectivity of rodent attack analysis and effectively accelerate the improvement of relevant research levels. Aggressive behavior score = the number of attacks + 0.2 × attack duration (s) + the number of bites + 0.2 × on-top duration (s) + piloerection [52]. The aggressive behavior was evaluated using the blind method, and the video was played back by three persons with uniform training. The results were recorded. The consistency test revealed that kappa > 0.95.

#### Open field test (OFT)

The rats were adapted to the test room for more than 10 min [56]. Each rat was then placed in an open field box (100cm × 100cm × 60cm), and the movement track of the rat was recorded with a camera for 5 min. The total distance was calculated using SuperMaze software (Softmaze, Shanghai, China). Before the next test, the open field box was cleaned with 75% alcohol. During the experiment, the experimenters were far away from the open field box.

#### Sucrose preference test (SPT)

In the SPT, one bottle of sucrose water and another bottle of 0.8% sucrose solution were allowed for each Wistar rat freely for 24 h [57]. After 12 h, the positions of the two bottles were switched to reduce the influence of side deviation. The bottles were weighted before and after the test. The sucrose preference coefficient was calculated based on the percentage of sucrose intake in liquid intake. For the control group, the rats were temporarily reared alone during the test period, then returned to their original cages immediately after the test.

### Immunofluorescence

#### Tissue processing

The rats were anesthetized with 75 mg/kg of sodium pentobarbital intraperitoneal injection (Sigma-Aldrich, UK). After deep anesthesia, a midline sternal incision was made to expose the heart, which was then perfused through the left ventricle. First, a quick infusion of normal saline 200 mL, and then 200 mL of 4% paraformaldehyde (Damao Chemical Reagent Factory, China) was perfused, first fast and then slow. The brain was completely removed and fixed with 4% paraformaldehyde for 2 h and transferred in 10%, 20%, and 30% sucrose solution in order of gradient transfer. After being completely dehydrated, the tissue was moved to cryostats (SLEE, Germany) at −20°C and then frozen in an OCT matrix (TissueTek, Sakura Finetek, UK) in sample preparation positions. Coronal sections of DRN (28 μm) (AP: −6.84~−8.40 mm) were placed into frozen stock solution (equal amounts of 0.01 M PBS and glycerin (Sinopharm Chemical Reagent Co., Ltd, China)) and stored at −20°C.

#### Immunofluorescence (5-HT and GB1)

The slices were fixed at room temperature for 30 min, washed six times 1x PBS for 5 min each, and then placed in 1% BSA blocking solution at room temperature for 60 min. The blocking solution was sucked up, and anti-GB1 antibody (1:1000, Abcam, USA, ab55051) and anti-5-HT antibody (1:1200, Abcam, USA, ab66047) were added to incubate for 1 h at room temperature and then overnight at 4°C. After washing three times in 1x PBS, the slices were incubated in the dark with secondary antibodies (rabbit anti-mouse IgG, 1:300, Abcam, USA, ab150125; rabbit anti-goat IgG, 1:300, Abcam, USA, ab6738) at room temperature for 2 h. The slices were washed again three times, placed on the slide and drop the anti-fade fluorescence mounting medium (Thermo Fisher Scientific, USA), and covered with the slide. After air-drying in the dark, the slides were observed using a confocal microscope (Zeiss, Germany).

#### IF intensity analysis

Zeiss Zen Lite 2012 was used for analysis. The DRN area was set to “Area of Interest”. According to the rat brain stereotaxic coordinates atlas (Paxinos and Watson, 1997), we analyzed the IF intensity of the DRN region (12 samples/group) in the same section. “Arithmetic mean intensity” was recorded as IF intensity.

### CGP35348 and baclofen microinjection

After behavioral testing, the rats were anesthetized with an intraperitoneal injection of 75 mg/kg pentobarbital sodium (Sigma-Aldrich, UK). After deep anesthesia, the rats were placed in a stereotactic device. The animal’s bilateral internal ear holes and incisors were fixed at three points using the flat-head fixation method. The incisors’ vertical position was adjusted to 3.3 ± 0.4 mm below the horizontal plane, and the front and rear fontanels were connected at the same horizontal height. The skin was cut approximately 1 cm along the midline of the skull, and the target point, DRN (AP: −7.8 mm, L: −2.0 mm, V: −6.3 mm, 20°C) was located on the skull surface with the brain store locator and drilled a hole. The DRN in each group was then microinjected with normal saline (0.2 μL), baclofen (0.2 μL, 1.5 mg/mL), or CGP35348 (1 μL, 20 mg/mL). The injection lasted for 2 min and the needle was left for 2 min. The skin was sutured and treated with gentamicin sulfate for anti-infection. Brain tissues (PFC, hippocampus, and hypothalamus) were extracted after 10 min.

### ELISA kits

The brain tissues were collected at 0, 1, 3, 5, and 7 days after treatment, and the PFC, hypothalamus, hippocampus, and DRN were rapidly separated, weighted, and stored at −20°C. Furthermore, brain tissue was frozen for 10 min after the microinjection of normal saline, baclofen or CGRP35348 into the DRN. The PFC, hippocampus, and hypothalamus were quickly separated from brain tissue on ice, weighed, and stored at −20°C. The brain tissue was rinsed and homogenized with PBS before being stored overnight at −20°C. The cell membrane was broken by freeze-thaw twice, centrifuged (5,000 × g, 4°C, 5 min), and determined immediately. 5-HT levels in the PFC, hippocampus, and hypothalamus; DRN (12 samples/group) were measured using ELISA kits (Cusabio, China) based on the manufacturer’s instructions. GABA levels in the DRN (12 samples/group) were measured using ELISA kits (RD, USA) based on the manufacturer’s instructions.

### Statistical analysis

All experiments were repeated two times. Graphpad Prism 6.0 was used for statistical analysis, and the results are expressed as means ± SEM (standard error of the mean). The data between the two groups were analyzed using the independent *t*-test. The normal distribution test was performed before the *t*-test. When there were more than two groups of data, one-way ANOVA was used. *P* < 0.05 was considered statistically significant.

## RESULTS

### Behavior tests

**Figure 2:**
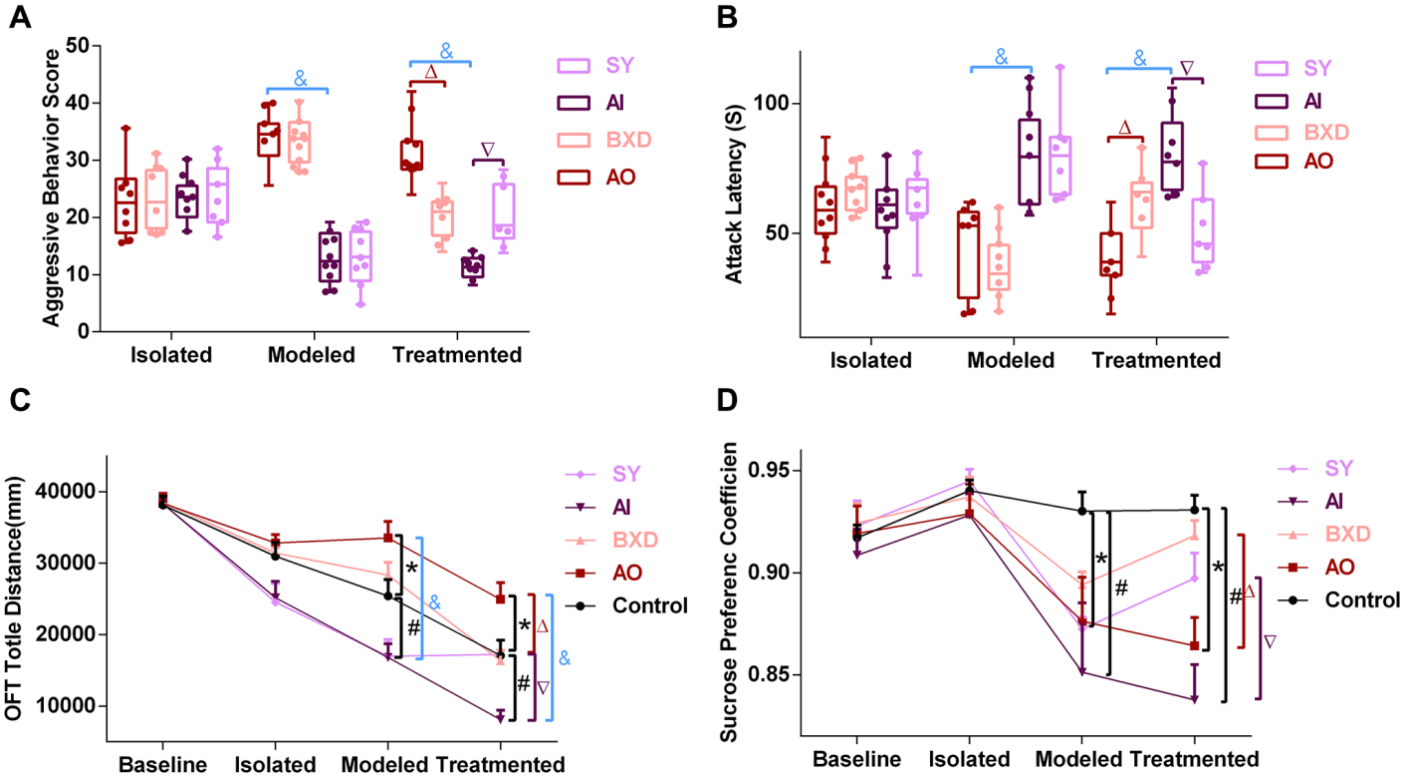
**Behavior tests.** (**A**) Aggressive behavior score, (**B**) Attack latency, (**C**) OFT total distance, (**D**) Sucrose preference coefficient; Data are expressed as means ± SEM, ^*^Anger-out group compared to the control group, ^#^Anger-in group compared to the control group, ^&^Anger-out group compared to Anger-in group, ^Δ^Anger-out group compared to the BXD group, ^∇^Anger-in group compared to the SY group; *P* < 0.05, *n* = 12.

Aggressive behavior score (ABS) is the main indication of the AO and AI rat models. As shown in Figure 2, the difference in ABS, attack latency (AL), OFT total distance, and sucrose preference coefficient (SPC) between groups at baseline were not statistically significant (*P* > 0.05). After modeling, rats in the group were divided into the AO and AI groups according to ABS and AL (Figures 2A, 2B); compared with the control group, the OFT total distance of the AO group significantly increased, whereas that of the AI group significantly decreased (*P* < 0.05) (Figure 2C). The SPC values of both the AO and AI groups were significantly lower than those of the control group (*P* < 0.05) (Figure 2D). After the medication, compared with the AO group, ABS and OFT total distances of the BXD group were all significantly decreased and AL was significantly increased (*P* < 0.05). Compared with the AI group, ABS and OFT total distances of the SY group were all significantly increased (*P* < 0.05), and AL was significantly decreased (*P* < 0.05). The SPC values of the BXD and SY groups were significantly increased (*P* < 0.05) and returned to the level of the control group. Moreover, a significant difference was observed between the AO and AI groups (*P* < 0.05).

### The basis for the selection of drug treatment days

**Figure 3:**
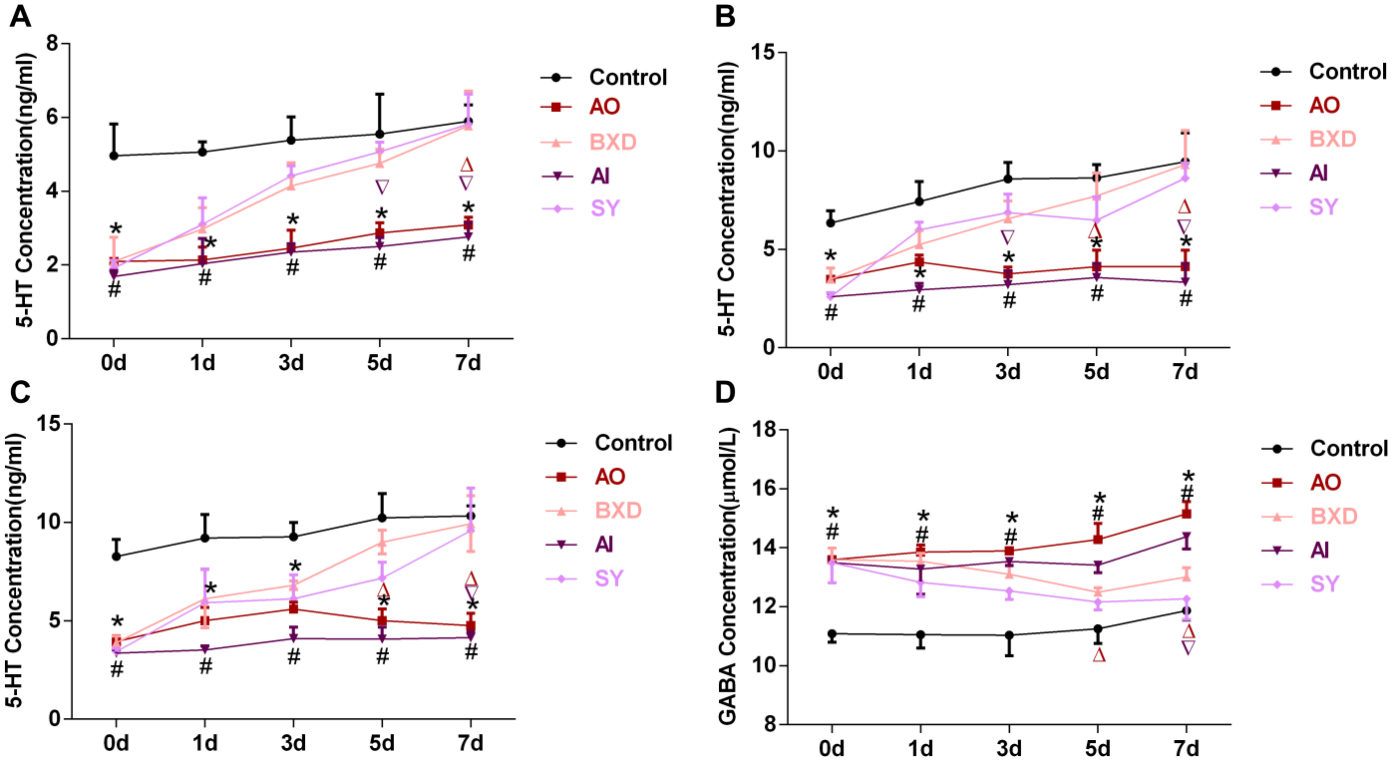
**5-HT concentration in the PFC, hippocampus and hypothalamus, and GABA concentration in the DRN after different days of treatment.** (**A**) 5-HT concentration in the PFC, (**B**) 5-HT concentration in the hippocampus, (**C**) 5-HT concentration in hypothalamus, (**D**) GABA concentration in DRN; Data are expressed as means ± SEM, ^*^Anger-out group compared to the control group, ^#^Anger-in group compared to the control group, ^&^Anger-out group compared to Anger-in group, ^Δ^Anger-out group compared to the BXD group, ^∇^Anger-in group compared to the SY group; *P* < 0.05, *n* = 8.

**Figure 4:**
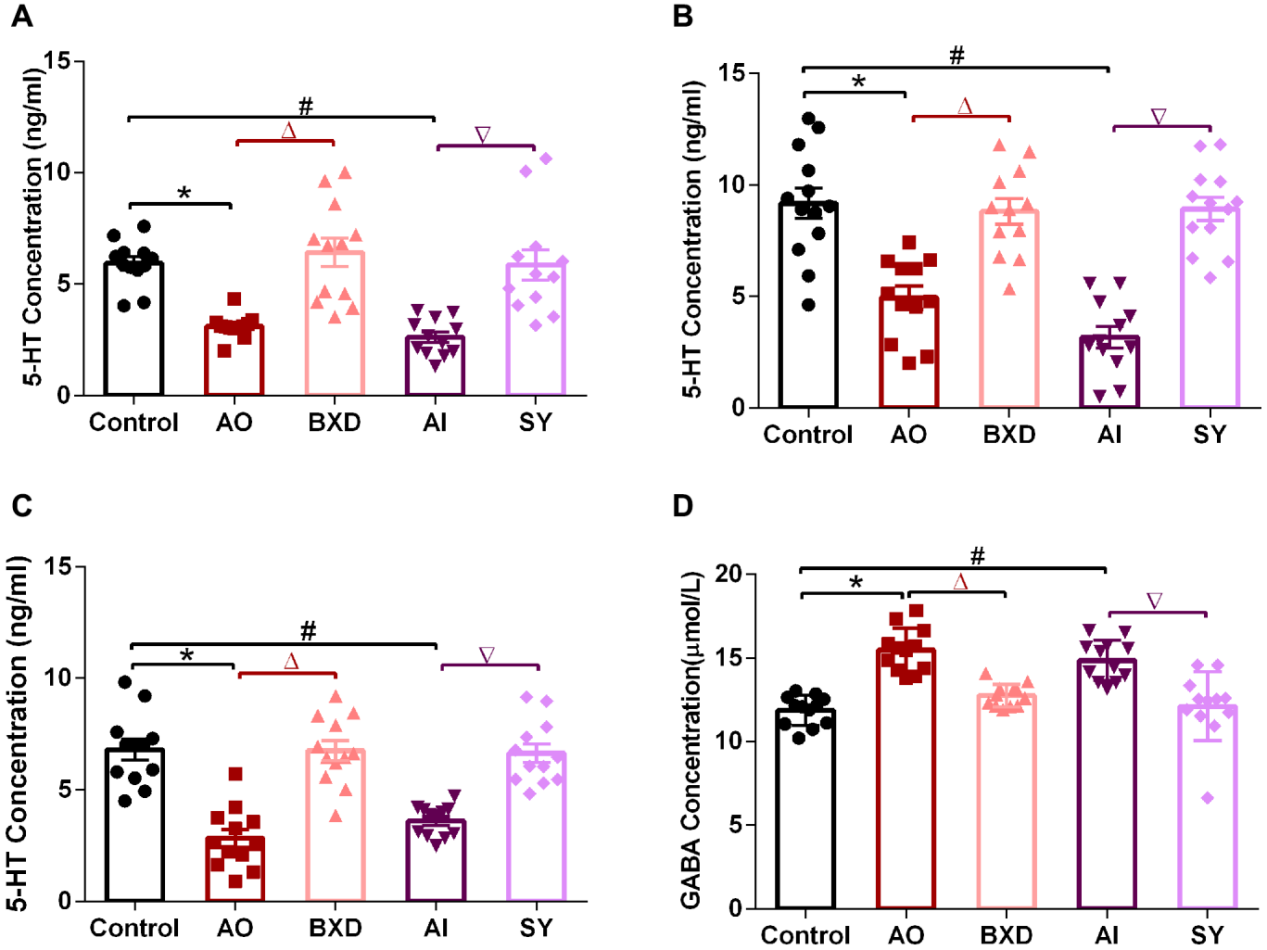
**5-HT concentration in the PFC, hippocampus and hypothalamus, and GABA concentration in DRN after 7 days of treatment.** (**A**) 5-HT concentration in the PFC, (**B**) 5-HT concentration in the hippocampus, (**C**) 5-HT concentration in hypothalamus, (**D**) GABA concentration in DRN; Data are expressed as means ± SEM, ^*^Anger-out group compared to the control group, ^#^Anger-in group compared to the control group, ^&^Anger-out group compared to Anger-in group, ^Δ^Anger-out group compared to the BXD group, ^∇^Anger-in group compared to the SY group; *P* < 0.05, *n* = 12.

As the model group received no drug intervention, the level of 5-HT in the PFC, hypothalamus, and hippocampus of the AO and AI groups was significantly lower than that of the control group (*P* < 0.05). By contrast, the GABA level in the DRN of the AO and AI groups was higher than that of the control group (*P* < 0.05). After the drug intervention, with the increase of the medication duration, the 5-HT levels in the PFC, hypothalamus, and hippocampus of the BXD and SY groups increased (*P* < 0.05), gradually corrected their abnormal decline, and returned to the normal level on the 7th day (Figures 3A–3C and 4A–4C). By contrast, the GABA level in the DRN decreased with the increase in medication duration (*P* < 0.05), gradually corrected its abnormal increase, and returned to normal level on the 7th day (Figures 3D, 4D).

### Histologic association of GB1 and 5-HT neurons in the DRN

#### IF (GB1 and 5-HT neuron in the DRN)

**Figure 5:**
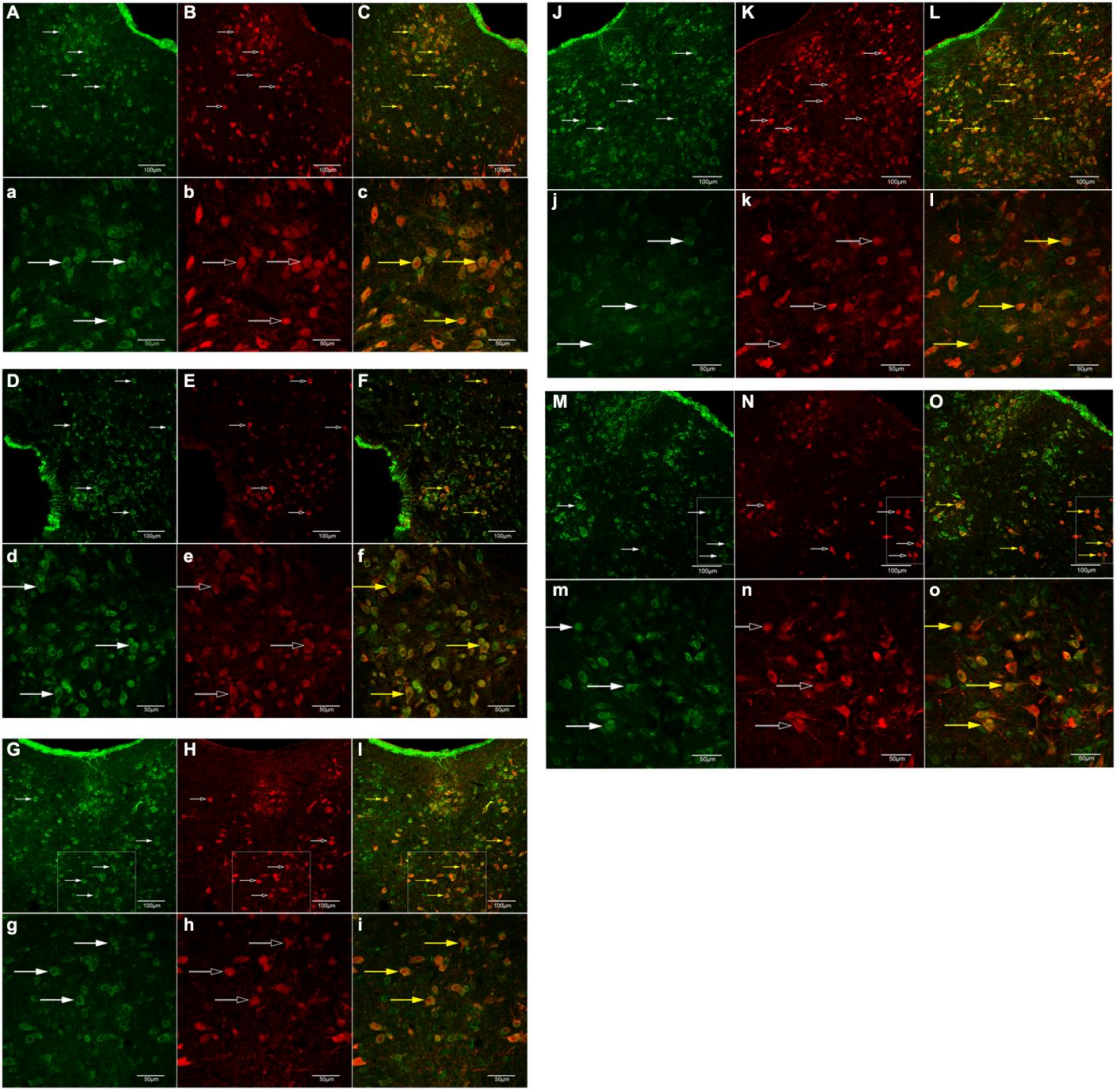
**IF of GB1 and 5-HT neurons in the DRN.** (**A**, **a**), (**B**, **b**), (**C**, **c**): control group; (**D**, **d**), (**E**, **e**), (**F**, **f**):AOM group; (**G**, **g**), (**H**, **h**), (**I**, **i**): BXD group; (**J**, **j**), (**K**, **k**), (**L**, **l**): AIM group; (**M**, **m**), (**N**, **n**), (**O**, **o**): SY group. (**A**, **a**), (**D**, **d**), (**G**, **g**), (**J**, **j**) and (**M**, **m**): green-labeled GB1; (**B**, **b**), (**E**, **e**), (**H**, **h**), (**K**, **k**) and (**N**, **n**): red-labeled 5-HT; (**C**, **c**), (**F**, **f**), (**I**, **i**), (**L**, **l**) and (**O**, **o**): double-labeled cells (yellow). (**A**–**O**): x200, scale = 50 mm; (**a**–**o**): x400, scale = 20 mm.

Previous studies have demonstrated that 5-HT neurons in the DRN have nerve fiber connections with the PFC, hypothalamus, and hippocampus [37]. Consequently, the expression and nerve connection of 5-HT neurons and GB1 in the DRN, as well as the nerve fiber connection of BXD (regulates AO) and SY (regulates AI). As shown in Figure 5, 5-HT immunostaining positive cells (red) (5-HT neurons) in the DRN of each rat group exhibited GB1 immunostaining positive cells (green). Thus, 5-HT and GB1 are co-expressed in the DRN and have a nerve fiber connection. To clarify the neuromorphological basis of BXD and SY in the treatment of AO and AI.

#### IF intensity value of GB1 and 5-HT

**Figure 6:**
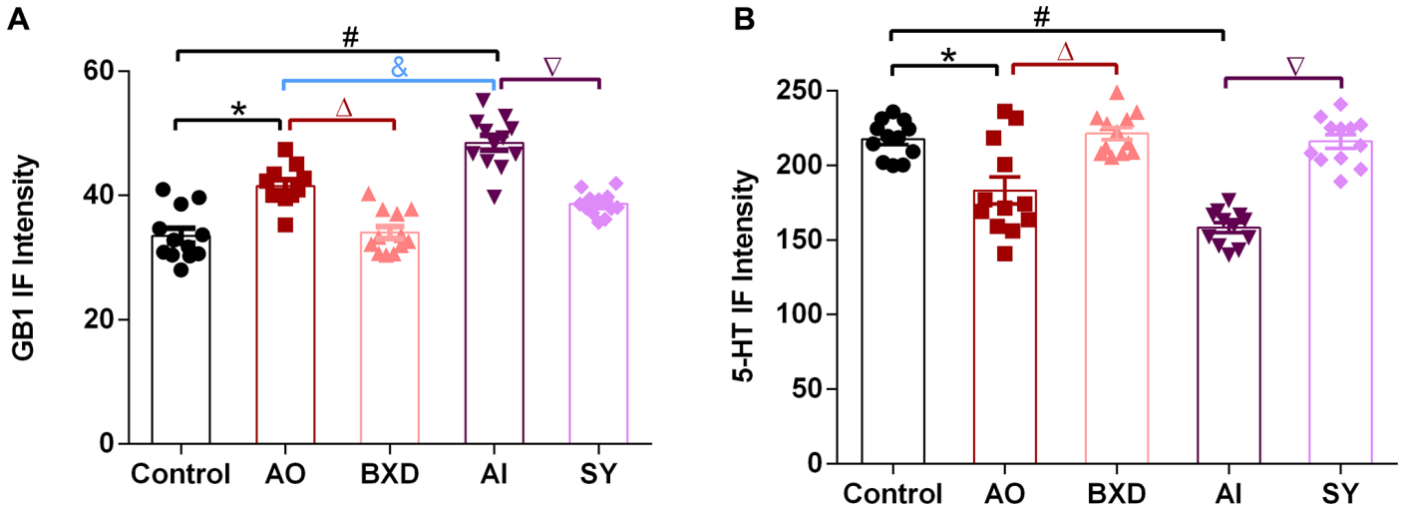
**IF intensity of GB1 and 5-HT in the DRN.** (**A**) GB1 intensity value, (**B**) 5-HT intensity value. Data are expressed as means ± SEM, ^*^Anger-out group compared to the control group, ^#^Anger-in group compared to the control group, ^&^Anger-out group compared to Anger-in group, ^Δ^Anger-out group compared to the BXD group, ^∇^Anger-in group compared to the SY group; *P* < 0.05, *n* = 12.

We performed a semi-quantitative analysis of immunofluorescence intensity. The results demonstrated that intensity in both model groups decreased significantly more than that in the control group (*P* < 0.05). Besides, after the drug intervention, 5-HT content in the DRN was elevated to normal levels in the BXD and SY groups (*P* < 0.05) (Figure 6A). However, the intensity in both model groups was increased significantly more than that in the control group (*P* < 0.05). Besides, the AI group increased significantly more than the AO group (*P* < 0.05). After the drug intervention, GB1 content in the DRN was decreased to normal levels in the BXD and SY groups (*P* < 0.05) (Figure 6B).

### 5-HT levels in PFC, hippocampus, and hypothalamus after microinjection of baclofen or CGP35348 into the DRN

**Figure 7:**
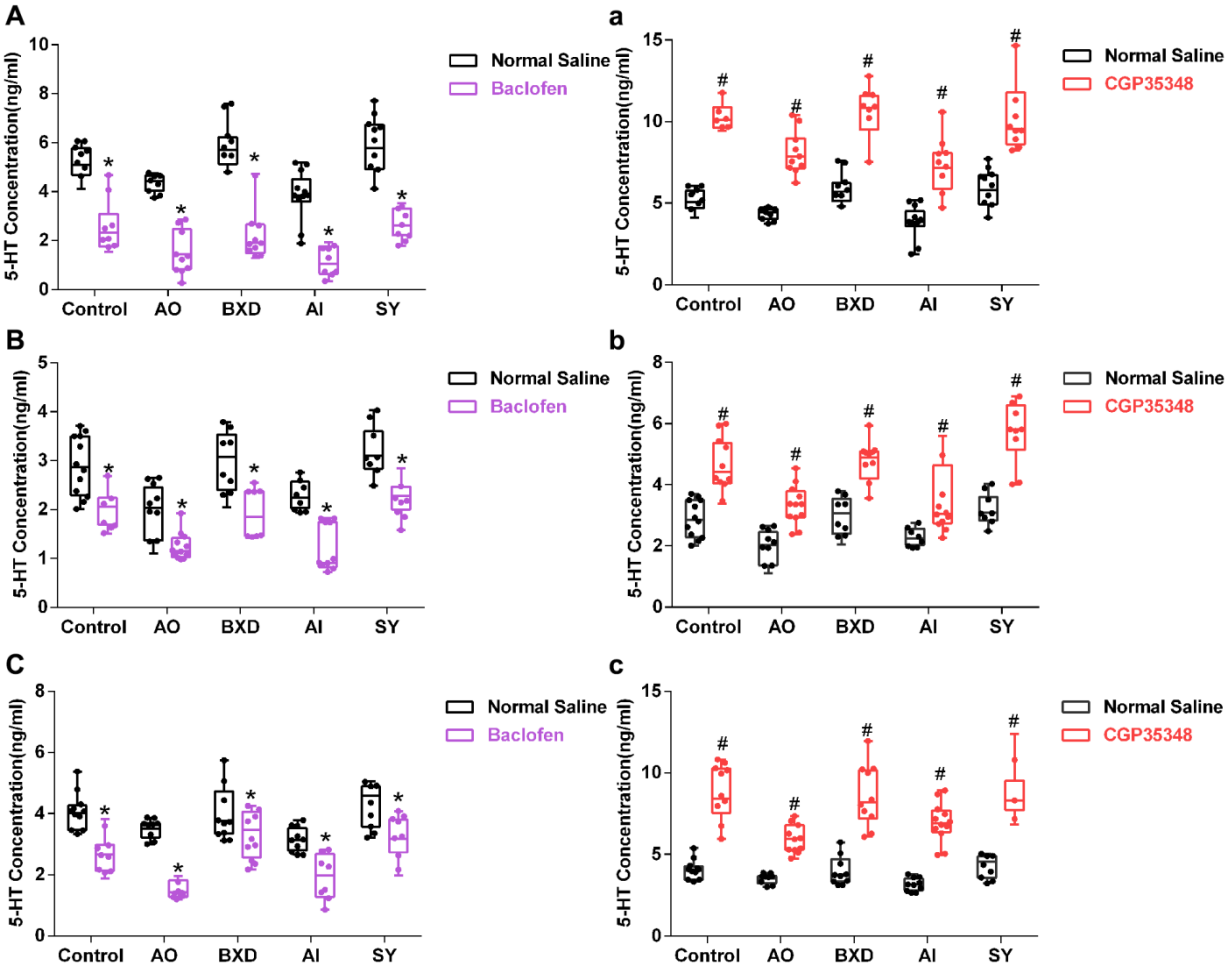
**The concentration of 5-HT in the PFC, hippocampus and hypothalamus after microinjection of baclofen or CGP35348 into the DRN.** (**A**, **a**): 5-HT concentration in the PFC, (**B**, **b**): 5-HT concentration in the hippocampus, (**C**, **c**): 5-HT concentration in hypothalamus. (**A**–**C**) microinjection of baclofen; (**a**–**c**) microinjection of CGP35348. Data are expressed as means ± SEM. ^*^Microinjection of baclofen compared with normal saline, ^#^Microinjection of CGP35348 compared with normal saline; *P* < 0.05, *n* = 12.

Finally, baclofen or CGP35348 was microinjected into the DRN to observe the changes in 5-HT levels in the PFC, hypothalamus, and hippocampus. As shown in Figure 7, the 5-HT concentration in the PFC, hippocampus, and hypothalamus of each rat group (Figure 7A–7C) was reduced significantly after baclofen– a specific agonist of GB1 (*P* < 0.05)–was microinjected, whereas the 5-HT concentration in the PFC, hippocampus, and hypothalamus in each rat group (Figure 7A–7C) was significantly increased after microinjection of CGP35348, a specific inhibitor of GB1 (*P* < 0.05).

## DISCUSSION

### AO and AI Rat

Social isolation combined with the resident-intruder paradigm is a classic modeling method in the rat model of AO and AI [46, 47]. Social isolation can induce aggressive behavior in rats [58]. The prolonged isolation of resident rats makes them irritable and aggressive. They can instinctively attack invaders, to protect their territory and generate hostile psychology and behavior. Previous studies have reported that social isolation combined with the resident-intruder paradigm could successfully prepare a scientific and stable rat model of high-aggressive and low-aggressive behavior [37]. Meanwhile, AO and AI rat models were successfully replicated in this paper, laying the foundation for subsequent experiments. However, part of the aggression is because rats, like humans, are a social species. Therefore, isolation-induced aggression likely has a different etiology than resident-intruder aggression and other types of aggression expressed in humans (e.g., rage and impulsiveness,). This may be a flaw in the current study.

Aggression or aggressive behavior, which may be triggered by anger [59], has various associations with anger. Aggression may also be viewed as the behavioral expression of anger [60]. Both anger and aggression could be seen as strong and lasting personal traits [61, 62], individuals with characteristics of aggression are often accompanied by a trait of anger and are more likely to generate anger and enmity in the face of offensive or irritating events [63]. Anger is characterized by increased physiological arousal, and an increased predisposition toward aggressive behavior [64, 65]. In the close association between anger and aggression, many neurochemical and genomic studies have included Spielberger’s Anger-Hostility-Aggression as a research object, in addition to anger itself [66].

ABT is the main indication of the AO and AI rat models. OFT is often used to measure anxiety-like behavior in rats [67]. OFT and SPT testified that emotional changes induced aggressive behavior and the complexity of emotions in rats. The generation of anger is often accompanied by a certain degree of anxiety and depression [68, 69]. In our study, after modeling, AO rats exhibited higher scores of aggressive behaviors, shorter attack latency, and a higher total distance of OFT than AI rats. The SPC of AO and AI rats was significantly lower than that of the control group. After BXD treatment, the score of AO rats’ aggressive behavior was decreased, the latency of aggression was prolonged, the total distance of OFT was shortened, and the SPC was increased. After SY treatment, the score of attack behavior of AI rats was increased, the latency of attack was shortened, the total distance of OFT was prolonged, and the SPC was increased. This finding suggests that BXD can effectively correct the abnormal behavior of AO rats, and SY can effectively correct the abnormal behavior of AI rats. These three evaluation methods have been also used in previous studies on attack behavior evaluation [68, 69]. The results of the present study also confirmed that these three evaluation methods could effectively evaluate the rat model of AO and AI, that BXD could effectively improve abnormal AO tendencies, and that SY could effectively improve abnormal AI tendencies by changing the score of the ABT, OFT, and SPT, and that the intervention effect was good. This result is consistent with the results of relevant reports [70, 71].

### Possible action targets and mechanism of BXD against AO and SY against AI

Many studies have demonstrated that 5-HT is a key neurotransmitter that regulates anger attacks, and depression patients with anger are more prone to 5-HT imbalance than patients without anger [34, 37, 72, 73]. Decreased levels of 5-HT or 5-HIAA in brain tissue can increase the occurrence of aggressive behavior and violence [74]. The activity of 5-HT neurons in the DRN increased aggressive behaviors among male mice [75]. By contrast, studies have also found that overexpression of 5-HT1A receptors reduces the activity of 5-HT neurons in the DRN and enhances the aggressive behavior of mice [76]. Studies in knockout mice have also reported that increased aggressive behavior was accompanied by decreased 5-HT levels or reduced activity [77–79]. Some scholars have revealed that different basal levels of 5-HT and phasic changes may have a different roles in different types of aggression [80]. In our study (Figure 5B), both AO and AI rats have a lower level of 5-HT expression in the DRN than normal rats, and AI rats have a lower level of 5-HT expression than AO rats. Furthermore, BXD and SY could treat the abnormally decreased 5-HT level in the DRN.

The PFC, hypothalamus and hippocampus are the key brain areas involved in emotion regulation [81, 82]. The cell body of 5-HT neurons is mainly in the DRN. The DRN has nerve fiber projections with the PFC, hippocampus, and hypothalamus [83], as evidenced by our preliminary study [37]. The results revealed that in AO and AI rats, 5-HT levels in the PFC, hypothalamus, and hippocampus were decreased in tandem with those in the DRN. Our previous research on high and low aggression yielded similar results [37]. Similarly, BXD and SY could treat the abnormal decrease of 5-HT levels in the PFC, hypothalamus, and hippocampus (Figures 3A–3C), and then regulate AO and AI, which require 7 days to achieve a curative effect (Figure 2C).

The release of 5-HT from 5-HT neurons in the DRN was significantly decreased in AO and AI rats, as were the levels of 5-HT projected to the PFC, hypothalamus, and hippocampus via fibers. BXD could relieve AO and SY could relieve AI by treating the aforementioned abnormalities.

GABA is an essential inhibitory neurotransmitter [39]. GABA can seriously affect the function of the PFC [84] and participate in the regulation of emotional disorders [40]. 5-HT and GABA levels have synaptic connections and regulate each other [37, 85–87]. Various pathological processes, including chronic pain, epilepsy, and schizophrenia, are often accompanied by changes in GB1 expression and function [88, 89]. Studies have confirmed that GABA can regulate the level of 5-HT in the DRN through GABABR and GABAAR [90]. The decreased expression of GB1 and GABABR2 in the hippocampus of rats may be related to the generation of AO, and the central mechanism of BXD for calming the liver and regulating qi may be related to the recovery of the expression and function of GABABR in the hippocampus [91]. BXD can also increase the abnormally decreased GABA in the cerebral cortex [92]. Depression model rats can induce the up-regulation of GABABR2 protein expression in rat hippocampal neurons. SY may play an antidepressant role by reducing the expression of GABABR2 protein in hippocampal neurons [93]; SY may also inhibit the decrease of the ratio of Glu to GABA in the hippocampus [94]. In this study, we found that GABA concentration in the DRN was increased and GB1 expression was up-regulated, BXD and SY could effectively reduce GABA concentration (Figure 3D) and down-regulate the expression of GB1 (Figure 5B), then regulate the AO and AI, with a 7-day treatment period required to achieve the curative effect (Figure 2D). This finding suggests that BXD can improve AO by reducing the GABA content in the DRN and down-regulating the expression of GB1, and the best time for improvement is 7 days after treatment. SY can also improve AI in the same way. This finding is consistent with that of relevant research. The intervention of BXD and SY could significantly reduce the scores of anger and depression expression and regulate the contents of 5-HT and GABA in rhesus monkeys [95]. SY can correct behavioral abnormalities and GABA abnormalities in the forehead, hypothalamus, and hippocampus in depressed rats [96]. However, some research results demonstrate that [97] the GABA content in each brain area of rhesus monkeys with liver-qi depression syndrome induced by anger is significantly reduced, whereas the GABA content in each brain area of rhesus monkeys with liver-qi depression syndrome induced by depression is significantly increased. This result is not consistent with that of this study. There may be differences in the models, and the changes in their internal biological indicators and mechanisms are not completely consistent. Furthermore, the expression of GB1 and GABABR2 in the hippocampus neurons of AO rats has decreased [91], which is negatively correlated with the results of the present study because the expression of GB1 is different due to different detected brain regions.

Furthermore, the results of the current study suggested that the GB1 immunoreactivity was increased in both AI and AO compared with that of control rats, whereas, the GB1 immunoreactivity in AI rats was higher than that in AO rats. However, for 5-HT immunoreactivity, no difference was found between AI and AO rats, suggesting that GB1 change is more sensitive to the main difference between AI and AO. The GABA terminal is in close contact with GABABR/5-HT double-standard neurons [37, 98]; GABA may regulate the activity of 5-HTergic neurons through GABABR and participate in the regulation of nociceptive information transmission [98]. The expression of GB1 in 5-HT neurons in the DRN of rats from each group was confirmed using the immunofluorescence double-labeling technique (Figure 4). We determined the objective conditions for GB1-mediated GABA regulation of the existence of 5-HT neurons in the DRN. Although having more the brain regions of histogram and fluorogram will provide more intuitive and in-depth insights, we have obtained the fluorogram and histogram of 5-HT and GB1 in key brain regions (Figures 5, 6), which could explain the problem. Of course, if conditions permit, adding more histograms and fluorograms of brain regions will be more perfect, providing a good guide for our future research work.

Therefore, GABA levels were increased significantly in the DRN of angry-out and AI rats, which further mediated the synaptic effect and exerted an inhibitory function by binding with GB1 on 5-HT neurons in the DRN, thus reducing the release of 5-HT in the DRN and leading to a significant decrease of 5-HT in the PFC, hypothalamus, and hippocampus. This is also the intervention target and way of BXD and SY.

Baclofen is a GB1-specific agonist (Ali Shah et al., 2013), which can bind to GB1 [99]; whereas CGP35348 is a GABABR-specific inhibitor that can specifically bind to GABABR on a presynaptic membrane and/or postsynaptic membrane. GABABR activation in the DRN would enhance the aggressive behavior of male mice, and microinjection of baclofen into the DRN could increase the level of extracellular 5-HT in the prefrontal region [75]. Baclofen can promote the expression of GB1, improve the binding ability of GABA and its receptor, enhance the inhibitory effect of GABA, and thus inhibit the release of 5-HT in the DRN [100]. The current study demonstrated that the levels of 5-HT in the PFC, hippocampus, and hypothalamus in each group were decreased significantly when the DRN was injected with baclofen, while the reverse was observed after the DRN was injected with CGP35348. Therefore, from both positive and negative aspects, GB1-mediated GABA regulates 5-HT levels in the PFC, hippocampus, and hypothalamus, which plays an essential role in AO and AI, and is also the intervention target of BXD and SY.

In conclusion, BXD and SY can act on GB1 in the DRN, and then affect the effect of GABA, thus affecting the levels of 5-HT in the PFC, hypothalamus, and hippocampus of AO and AI rats. Thus, GB1 in the DRN mediates GABA regulation of 5-HT levels in the PFC, hypothalamus, and hippocampus, which is the target of BXD in regulating AO and SY in regulating AI. However, some scholars [101] have found that the extracellular 5-HT content of the medial prefrontal cortex increased after microinjection of baclofen into the DRN, which may be because the total content of 5-HT in and out of the cells in the prefrontal region was not synchronized with the change in extracellular 5-HT content. Furthermore, baclofen injection into the DRN at night can decrease the level of 5-HT in the DRN [100], whereas daytime injection can increase the level of 5-HT in the DRN [102]. Therefore, different injection times of baclofen may lead to the opposite expression of 5-HT.

Notably, BXD can correct the abnormal GB1-mediated GABA regulation of 5-HT levels in the PFC, hypothalamus, and hippocampus in AO, whereas SY can correct the aforementioned changes in AI, and the correction direction is the same. However, BXD can correct the abnormal behavior in AO rats, and SY can correct the abnormal behavior in AI rats, but the correction direction is the opposite. BXD is composed of Paeoniflorin, the volatile oil of Rhizoma Cyperi, and Paeonol, the main effective component of *Paeonia lactiflora pall*, *Rhizoma Cyperi*, and *Cortex moutan*. SY is composed of Bupleurum saponin, Paeoniflorin, and volatile oil of Cyperus, the main effective component of *Radix Bupleuri, Radix Paeoniae Alba, and Rhizoma Cyperi.* Thus, both BXD and SY contain paeoniflorin and the volatile oil of Rhizoma Cyperi. Paeoniflorin could ameliorate symptoms and improve the functional capability of post-stroke depression rats [103], and it can also protect against cognitive impairment [104]. The volatile oil of Cyperus rotundus can inhibit depression by regulating the content of 5-HT in the brain [105]. However, BXD contains paeonol, whereas SY contains Bupleurum saponin. Paeonol is capable of not only calming hypnosis but also significantly improving learning memory and anxiety [106]. Saikosaponin can increase the 5-HT level in rat brain, and effectively improve depression-like behavior [107]. Saponin A can improve depression-like behavior and regulate the expression of 5-HT and NE in the hippocampus of rats [108]. Paeoniflorin and Cyperus volatile oil may be inclined to correct the micro mechanism of action; Paeonol may be inclined to behavioral correction of anger; Bupleurum saponin may be inclined to behavioral correction of depression. These findings provide an indication for further exploration in the future.

As basic research, the results provide a new scientific basis for in-depth exploration of the brain’s central mechanism of “AO and AI”, clarify the respective corresponding action targets of BXD and SY, and thus open up new ways and new ideas for revealing the essence of Traditional Chinese Medicine (TCM) theory “anger harms the liver”, as well as for basic research and clinical treatment of TCM emotional diseases.

## CONCLUSIONS

BXD could effectively improve the abnormal behavior changes of AO rats, SY could effectively improve the abnormal behavior changes of AI rats, and the optimal duration of action was 7 days. GB1 was increased differentially in AI and AO rats and was alleviated by both BXD and SY. The activation or antagonism of GB1 in the DRN could affect the 5-HT concentration of the PFC, hippocampus, and hypothalamus. The ways for BXD to improve AO and SY to improve AI are as follows: Decreased abnormal increase of GABA and GB1 in DRN further mediated synaptic inhibition, and increased 5-HT level in the DRN, leading to increased 5-HT levels in the PFC, hypothalamus, and hippocampus. Therefore, GB1-mediated GABA could regulate the 5-HT levels in DRN, as well as 5-HT levels in the PFC, hypothalamus, and hippocampus, which may be one of the ways by which BXD and SY treat AO and AI, respectively.
